# The expression signature of very long non-coding RNA in myalgic encephalomyelitis/chronic fatigue syndrome

**DOI:** 10.1186/s12967-018-1600-x

**Published:** 2018-08-17

**Authors:** Chin-An Yang, Sandra Bauer, Yu-Chen Ho, Franziska Sotzny, Jan-Gowth Chang, Carmen Scheibenbogen

**Affiliations:** 1 Department of Laboratory Medicine, China Medical University Hospital, Taichung, Taiwan; 2 Division of General Pediatrics, Children’s Hospital of China Medical University, Taichung, Taiwan; 3 College of Medicine, China Medical University, Taichung, Taiwan; 4 Center for Precision Medicine, China Medical University Hospital, Taichung, Taiwan; 5 Institute for Medical Immunology, Charité-Universitätsmedizin Berlin, Augustenburger Platz 1, 13353 Berlin, Germany

**Keywords:** Myalgic encephalomyelitis/chronic fatigue syndrome, Long non-coding RNA, Expression signature, Peripheral blood mononuclear cells

## Abstract

**Background:**

Myalgic encephalomyelitis/chronic fatigue syndrome (ME/CFS) is a chronic debilitating disease with huge social-economic impact. It has been suggested that immune dysregulation, nitrooxidative stress, and metabolic impairment might contribute to disease pathogenesis. However, the etiology of ME/CFS remains largely unclear, and diagnostic/prognostic disease markers are lacking. Several long noncoding RNAs (lncRNA, > 200 bp) have been reported to play roles in immunological diseases or in stress responses.

**Methods:**

In our study, we examined the expression signature of 10 very long lncRNAs (> 5 kb, CR933609, His-RNA, AK124742, GNAS1-AS, EmX2OS, MIAT, TUG1, NEAT1, MALAT1, NTT) in the peripheral blood mononuclear cells of 44 ME/CFS patients.

**Results:**

LncRNAs NTT, MIAT and EmX2OS levels were found to be significantly elevated in ME/CFS patients as compared with healthy controls. Furthermore, NTT and EmX2OS levels increased with disease severity. Stimulation of human monocytic cell line THP-1 and glioma cell line KALS1 with H_2_O_2_ (oxidative stress) and poly (I:C) (double strand RNA, representing viral activation) increased the expression levels of NTT and MIAT.

**Conclusions:**

Our study revealed a ME/CFS-associated very long lncRNA expression signature, which might reflect the regulatory response in ME/CFS patients to oxidative stress, chronic viral infection and hypoxemia. Further investigations need to be done to uncover the functions and potential diagnostic value of these lncRNAs in ME/CFS.

## Background

Myalgic encephalomyelitis/chronic fatigue syndrome (ME/CFS) is a chronic debilitating disease with huge social-economic burden. The prevalence rate is 1–3/1000 in Germany and in the US population, affecting more than 1 million Americans [1]. In Taiwan, the estimated overall incidence rate of ME/CFS is 0.87–1.37/1000 person-year [2]. According to the Canadian criteria for diagnosis, ME/CFS patients suffer from more than 6 months of significant fatigue and post-exertional malaise, which describes the aggravation of symptoms upon exertion associated with sleep disturbance, myalgia/arthralgia, cognitive impairment, and autonomic dysfunction [3]. The pathogenesis of ME/CFS is complex, and remains mostly elusive so far. There is evidence that in a subset of patients infections result in chronic immune activation and autoimmunity [4]. Autoantibodies to neurotransmitters have been found in a subset of ME/CFS patients [5]. In addition, elevated cytokines and oxidative/nitrosative stresses possibly facilitate blood brain barrier disruption, neuroinflammation, and glial activation and hypersensitivity, which further triggers dysregulation of neurotransmitters and amplification of inflammatory signals [4]. More recently, mitochondrial dysfunction and metabolic disturbances have also been reported to underlie the potential mechanisms of ME/CFS [6, 7]. Despite these evidences, diagnostic markers are still lacking, and the diagnosis is usually made by clinical criteria.

Long non-coding RNAs are defined as RNAs that are more than 200 nucleotides long, which do not encode proteins. Through RNA–DNA, RNA–RNA, or RNA–protein interactions, lncRNA can affect different stages of gene regulation [8]. LncRNAs are key regulators of chromatin state, which show great capacity to interact with more than one protein in different context, and fine-tune the cellular response [8]. It has been reported that lncRNAs play essential roles in complex diseases, such as cancer and autoimmune diseases [9–11]. Although more and more lncRNAs are being discovered, most of their functions and mechanisms of actions are still unknown, especially for the very large lncRNAs that have sizes of more than 5000 nucleotides. We had particular interests in ten very large lncRNAs (> 5 kb), which have been either reported to be involved in immune regulation, or are located close to genes regulating stress response, metabolic and neurologic processes, thus potentially playing a role in ME/CFS.

The ten lncRNAs are NTT (17 kb), NEAT1 (23 kb), MALAT1 (7.5 kb), TUG1 (7.1 kb), MIAT (9.9 kb), His-1 RNA (8.4 kb), GNAS1-AS (8.9 kb), EMX2OS (7.3 kb), CR933609 (8.8 kb) and AK124742 (6 kb). NTT was first described in activated T cells, while NEAT1 has been reported to be involved in human lupus and in immune response to viral infections [11–13]. MALAT1 has been found to regulate LPS-induced inflammatory response, and TUG1 is involved in the regulation of cold-induced oxidative stress and inflammation [14–16]. As for MIAT, it is known to play roles in a variety of disease processes, including myocardial infarction, microvascular dysfunction, schizophrenia, and neurogenic commitment [17, 18]. His-1 RNA has been implicated in leukemogenesis, and GNAS1-AS is an imprinted anti-sense transcript at the locus of *GNAS1*, encoding neuroendocrine secretory protein [19, 20]. According to the lncrnadb database, EMX2OS is an opposite strand transcript of *EMX2* gene, and possibly regulates *EMX2* [21]. Both EMX2OS and EMX2 RNAs have been detected in the central nervous system (CNS) tissues [22]. For CR933609, we have previously identified its role in protecting INO80D from downregulation by miRNA-5096 [23]. Since INO80D is a main component of the chromatin re-modeler INO80 complex which regulates cell glycolytic and respiratory capacities, CR933609 could be involved in maintaining metabolic stability [24]. Finally, AK124742 has been reported to be an antisense RNA to the gene *PSMD6*, which encodes components of proteasome, involving antigen presentation by MHC class I and DNA damage repair [25, 26].

In this study, we aimed to investigate the expression signatures and potential diagnostic values of the ten very large lncRNAs (> 5 kb) in ME/CFS patients. Furthermore, the effects of oxidative stress (H_2_O_2_) and Toll like receptor 3 (TLR3) ligand poly (I:C) (mimicking viral infection) on the levels of very large lncRNAs were also evaluated in human monocytic cell line THP-1 and glioma cell line KALS1.

## Methods

### Subjects

Forty-four ME/CFS patients diagnosed according to the Canadian criteria and 30 sex and age-matched healthy controls were recruited. Characteristics of the study population are shown in Table 1. The study was approved by the Ethics Committee of Charité Universitätsmedizin Berlin in accordance with the 1964 Declaration of Helsinki and its later amendments and patients gave written informed consent.

**Table 1:** Characteristics of the study population

	Healthy control	ME/CFS patient	p value
Total, n	30	44	
Age (years)	37.3 ± 11.0	39.5 ± 10.9	0.18*
Sex			
Female	18 (60%)	28 (64%)	0.81^#^
Male	12	16	
Bell score			
≥ 30	NA	19	
< 30	NA	25	
Acute onset with infection	NA	39	
ME/CFS duration (years)	NA	7.1 ± 5.0	

* p value calculated by Mann–Whitney U test

^#^ p value calculated by Fisher’s test

### RNA extraction and RT-PCR for lncRNAs

RNAs were isolated from PBMCs using TRIzol Reagent (Thermo Fisher Scientific-Invitrogen, Waltham, Massachusetts, USA). High Capacity cDNA Reverse Transcriptase Kit (Thermo Fisher Scientific-Applied Biosystems, Waltham, Massachusetts, USA) was used to reverse-transcribe 2 μg RNA into cDNA using for real time PCR analyses. GAPDH expression was used as an endogenous control. All primers were designed and synthesized by Genomics BioSci & Tech, Taipei, Taiwan. The following primer sequences were used: *NTT* forward 5′-cttggcctaaaaggggatg-3′, reverse 5′-gcacctttggtctccttcac-3′; *MALAT1* forward 5′-gacccttcacccctcacc-3′, reverse 5′-ttatggatcatgcccacaag-3′; *TUG1* forward 5′-gtctccgatagtgcacacagc-3′, reverse 5′-gaccatctccttcaggacca-3′; *NEAT1* forward 5′-ctctgacccgaagggtagg-3′, reverse 5′-ctggcagctttgctcctg-3′; *MIAT* forward 5′-ctggagagggaggcatctaa-3′, reverse 5′- aactcatccccacccacac-3′; *His*-*1 RNA* forward 5′-cagtcttctttgaactgctactcct-3′, reverse 5′-tttttgtaacgctctggtcaaa-3′; *GNAS1*-*AS* forward 5′-ctgctatctgcagaggggtct-3′, reverse 5′-tcctttagctgcatttgct-3′; *EMX2OS* forward 5′-gtgacttgcacaaggacacaa-3′, reverse 5′-cctgtvtggccattcctct-3′; *CR933609* forward 5′-gacaaaacaaactagtgaagcacct-3′, reverse 5′-tatacaccttgacacggcaga-3′; *AK124742* forward 5′- aagttgtaggcatccttctagcc-3′, reverse 5′- cacttttgttaagcacccaactt-3′; *IFNGR1* forward 5′-catgcagggtgtgagcag-3′, reverse 5′-aacattagttggtgtaggcactga-3′; *PBOV1* forward 5′-gaaaaagattctcatcactcaac-3′, reverse 5′-ggttctcaaacagccttcc-3′; *GAPDH*: forward 5′-agccacatcgctcagacac-3′, reverse 5′-gcccaatacgaccaaatcc-3′.

### Cell line studies

Human monocytic cell line THP-1 and glioma cell line KALS1 were grown in LPS-free complete RPMI medium containing 10% fetal bovine serum, at 37 °C incubator with 5% CO_2_. Cells were treated with different concentrations of peroxide (H_2_O_2_) (Sigma Merk, Darmstadt, Germany) or poly (I:C) (InvivoGen, California, USA) for 6 h. RNAs were then extracted for further RT-PCR.

### Bioinformatics and statistical analysis

Principle component analyses were performed using Stata 13 software. Mann–Whitney U tests were used via GraphPad Prism 5 to analyze the difference of lncRNA expression levels between ME/CFS and controls. Receiver operating characteristic (ROC) curve analysis was performed using MedCalc v.14.

## Results

### PBMC lncRNA profile in chronic fatigue patients and controls

**Fig. 1:**
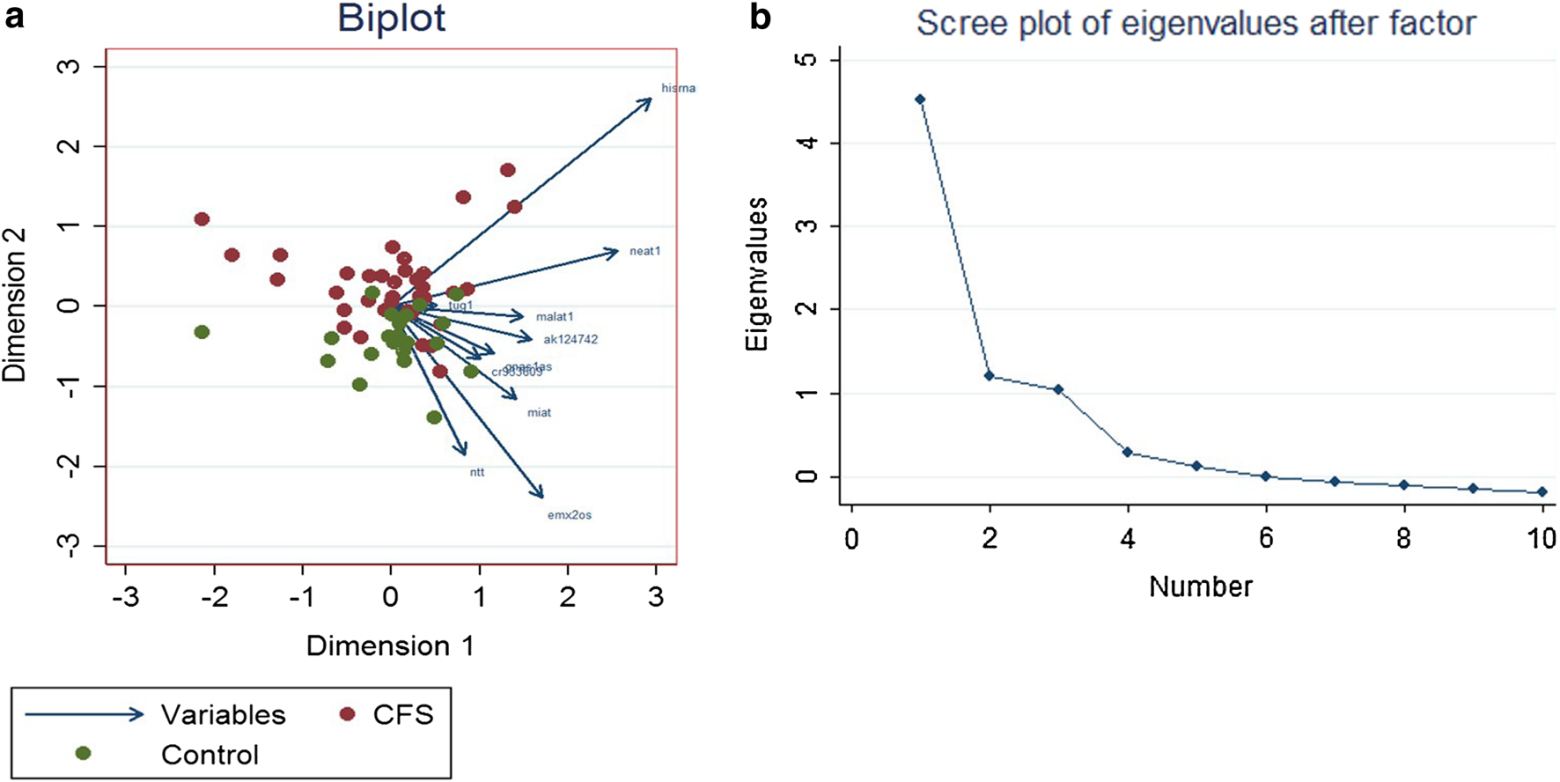
Principle component analysis (PCA) of ΔCT values of ten lncRNAs from PBMCs of ME/CFS patients and controls. **a** Variance between ME/CFS (red dots) and control (green dots) according to the ten lncRNA correlation matrix, displayed onto a two-dimension plot. **b** Eigenvalues of principle components

Expression of the ten lncRNAs (NTT, NEAT1, MALAT1, TUG1, MIAT, His-1 RNA, GNAS1-AS, EMX2OS, CR933609 and AK124742) in peripheral blood mononuclear cells (PBMCs) of ME/CFS patients and healthy controls were evaluated by RT-PCR. The values of the ten ΔCT (CT^lncRNA^–CT^GAPDH^) of each individual subject were used to build a correlation matrix, and projected to principle component space by principle component analysis (PCA) (Fig. 1a). The PCA showed that the ME/CFS and the control lncRNA expression profile could be separated mainly by principle component 2 (dimension 2). Similarly, the eigenvalues showed that data variances is mostly explained by the first and the second components (Fig. 1b).

### Association of lncRNA expression levels with ME/CFS and disease severity

**Fig. 2:**
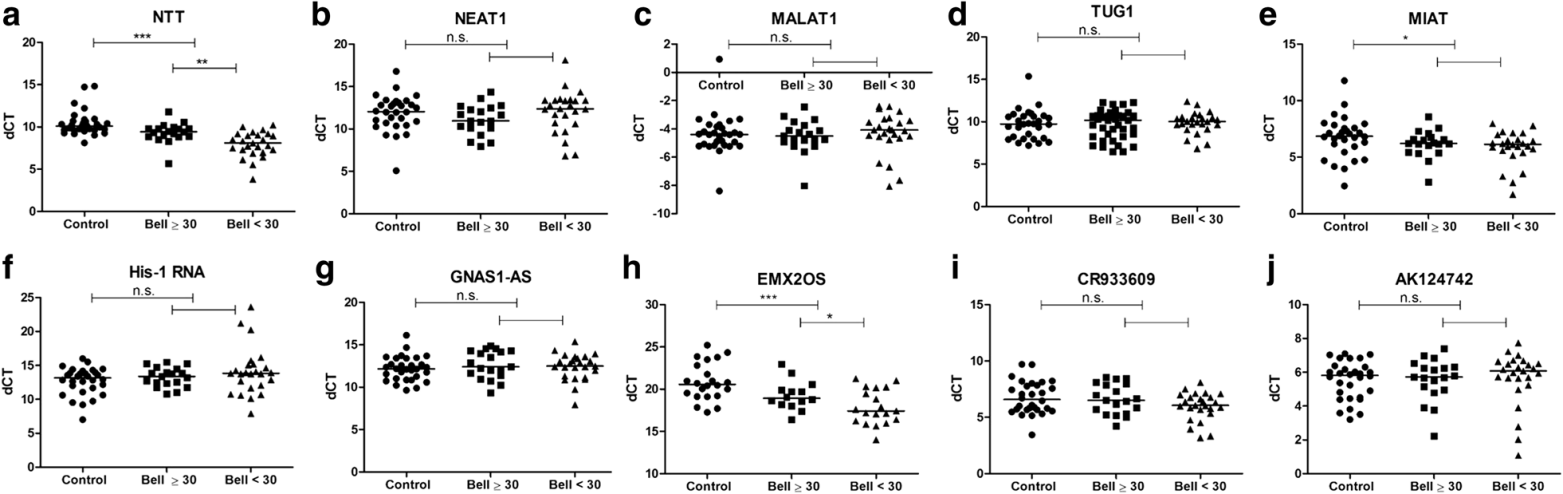
Expression of the ten very long lncRNA in PBMCs of ME/CFS and controls (**a**–**j**). ME/CFS patients with Bell score < 30 belong to the severe group. *p < 0.05, **p < 0.01, ***p < 0.001 by Mann–Whitney U tests. Lines represent medians. *ns* not statistically significant

In order to reduce the test numbers of lncRNAs needed for differentiating ME/CFS from healthy controls, and to evaluate the association of lncRNA expression level with ME/CFS disease severity, we analyzed the amount of each lncRNA in PBMCs of controls, mild-moderate ME/CFS (with Bell score ≥ 30), and severe ME/CFS (with Bell score < 30) (Fig. 2a–j). Interestingly, NTT, MIAT and EMX2OS levels were found to be significantly higher in ME/CFS PBMCs as compared with controls (ME/CFS median ΔCT vs. control median ΔCT: NTT 8.86 vs. 10.05, Mann–Whitney U test p < 0.0001; MIAT 6.22 vs. 6.89, Mann–Whitney U test p < 0.05; EMX2OS 20.69 vs. 18.59, Mann–Whitney U test p < 0.001). Furthermore, NTT and EMX2OS expression levels correlated with ME/CFS disease severity, with highest amount detected in ME/CFS patients with Bell score of 10 or 20 (< 30) (Fig. 2a, h).

### PBMC NTT, MIAT and EMX2OS expression signature as ME/CFS diagnostic markers

**Fig. 3:**
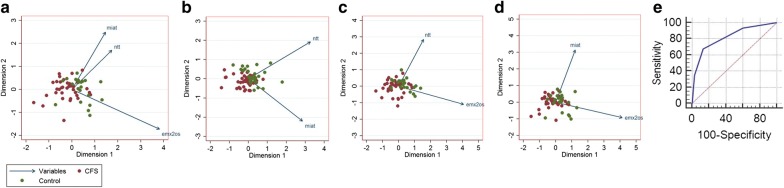
Diagnostic value of NTT, MIAT and EMX2OS expressions in ME/CFS. PCA showing the distribution of ME/CFS and controls on two-dimension plots differentiated by values derived from the expression levels of NTT, MIAT and EMX2OS (**a**) or from the expression levels of any two of the three lncRNAs (**b**–**d**). **e** Receiver-operating characteristic (ROC) curve analysis of the three-lncRNA signature to discriminate ME/CFS patients from healthy controls. AUC = 0.82 when the criteria is increased expression (ΔCT below the optimal cutoff) of any two of the three lncRNAs (NTT, MIAT and EMX2OS)

Since we have discovered that among the ten lncRNAs tested, only NTT, MIAT and EMX2OS levels were upregulated in ME/CFS, we plotted a new PCA plot using a correlation matrix of NTT, MIAT and EMX2OS ΔCT values (Fig. 3a). In Fig. 3a, ME/CFS patients could be separated from healthy controls by principle component 1 (dimension 1). The ME/CFS group could still be separated from the control group when the input lncRNA values were reduced to those of NTT and MIAT (Fig. 3b) or those of NTT and EMX2OS (Fig. 3c). However, the ME/CFS and the control group could not be well differentiated from each other when the PCA was performed on a correlation matrix of only MIAT and EMX2OS (Fig. 3d).

To evaluate the diagnostic value of the combination of the expression of NTT, MIAT and EMX2OS, we first obtained the optimal cutoff ΔCT value of each of the three lncRNAs by using receiver operating characteristic (ROC) curves. The optimal cutoff ΔCT value for NTT, MIAT and EMX2OS were 9.49 (area under the curve (AUC) = 0.82, 95% CI 0.72–0.90); 6.82 (AUC = 0.65, 95% CI 0.53–0.75); 19.06 (AUC = 0.77, 95% CI 0.64–0.87), respectively. Further ROC analysis was then performed and revealed that the criteria of increased expression (ΔCT below the optimal cutoff) of any two of these three lncRNAs in diagnosing ME/CFS had a sensitivity of 67.4, and specificity of 86.7, AUC = 0.82, with 95% CI 0.71–0.90 (Fig. 3e).

### Expression levels of genes potentially regulated by the lncRNAs in ME/CFS

**Fig. 4:**
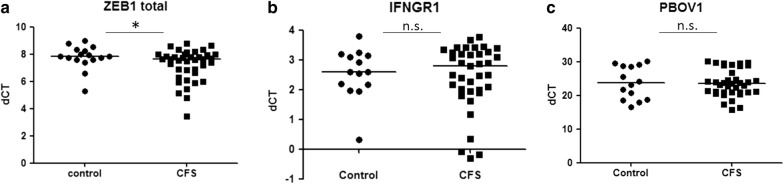
Expression level of genes potentially regulated by MIAT (**a**) or NTT (**b**, **c**) in PBMCs of ME/CFS patients and controls. *p < 0.05 by Mann–Whitney U test, lines represent medians

**Fig. 5:**
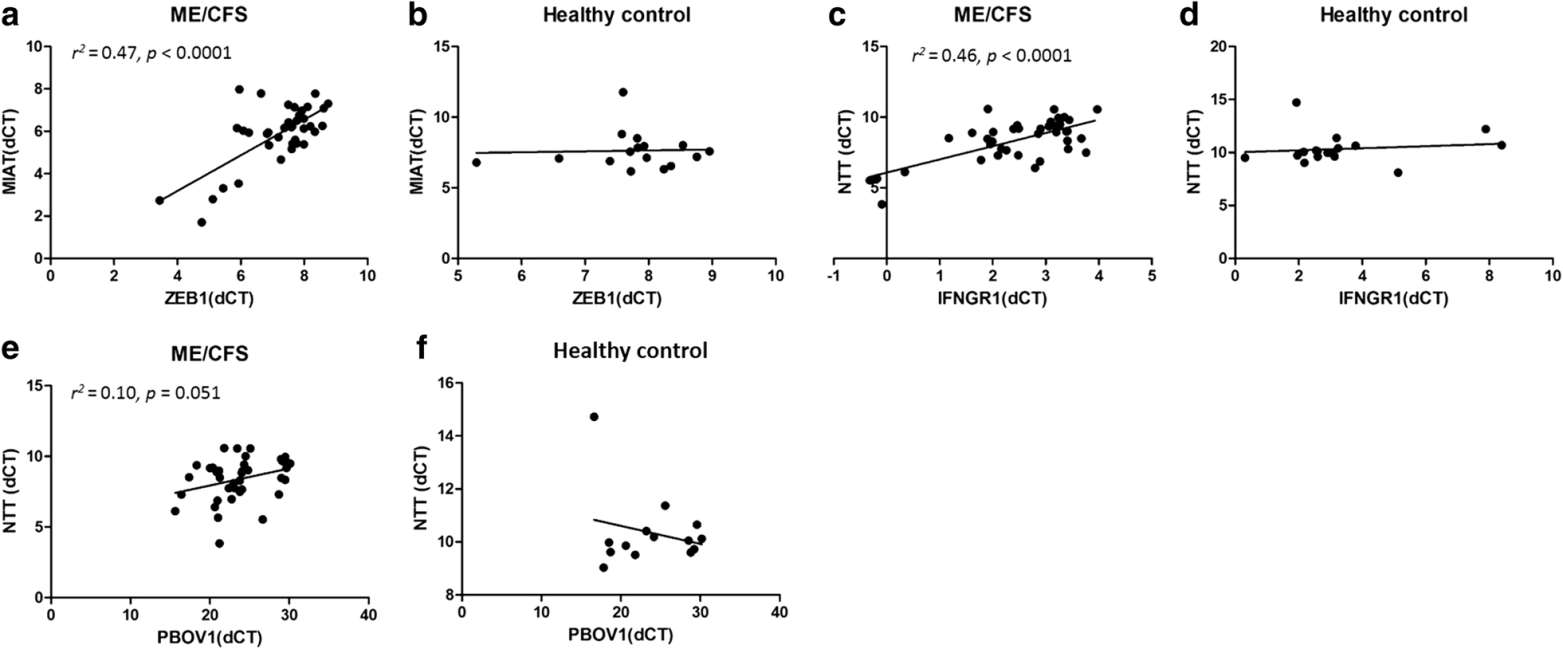
Correlation between lncRNA and downstream gene expressions. Correlation between MIAT and *ZEB1* levels in ME/CFS (**a**) and in controls (**b**). Correlation between NTT and *IFNGR1* in ME/CFS (**c**) and in controls (**d**). Correlation between NTT and *PBOV1* levels in ME/CFS (**e**) and in controls (**f**)

In order to elucidate potential mechanisms of actions of lncRNAs NTT, MIAT and EMX2OS in ME/CFS, we analyzed the expression profile of their potential downstream genes in patients and controls. NTT has been suggested to act on nearby genes, including *IFNGR1* and *PBOV1* [12]. *ZEB1* has been reported to be downstream of MIAT; and *EMX2* has been proposed to be regulated by EMX2OS [21, 27]. While *EMX2* could not be detected in PBMCs (data not shown), *ZEB1* level was found to have a mild but significant elevation in ME/CFS PBMCs as compared with controls (ME/CFS median ΔCT vs. control median ΔCT:7.60 vs. 7.82, p < 0.05, Fig. 4a). The expression levels of *IFNGR1* and *PBOV1* did not show significant differences between ME/CFS and controls (Fig. 4b, c). As for the correlation between the expressions of lncRNAs and downstream genes, a positive relationship between *ZEB1* and MIAT level was detected using linear regression analysis in ME/CFS (*r*^*2*^= 0.47, *p *< 0.0001), but not in controls (Fig. 5a, b). Similarly, *IFNGR1* level was found to be positively correlated with NTT level in ME/CFS (*r*^*2*^= 0.46, *p *< 0.0001), but not in controls (Fig. 5c, d). The linear regression analysis between NTT and *PBOV1* showed no statistical significance both in ME/CFS and controls (Fig. 5e, f).

### Upregulation of NTT and MIAT by H_2_O_2_ and poly (I:C) in human monocytic and glioma cell lines

**Fig. 6:**
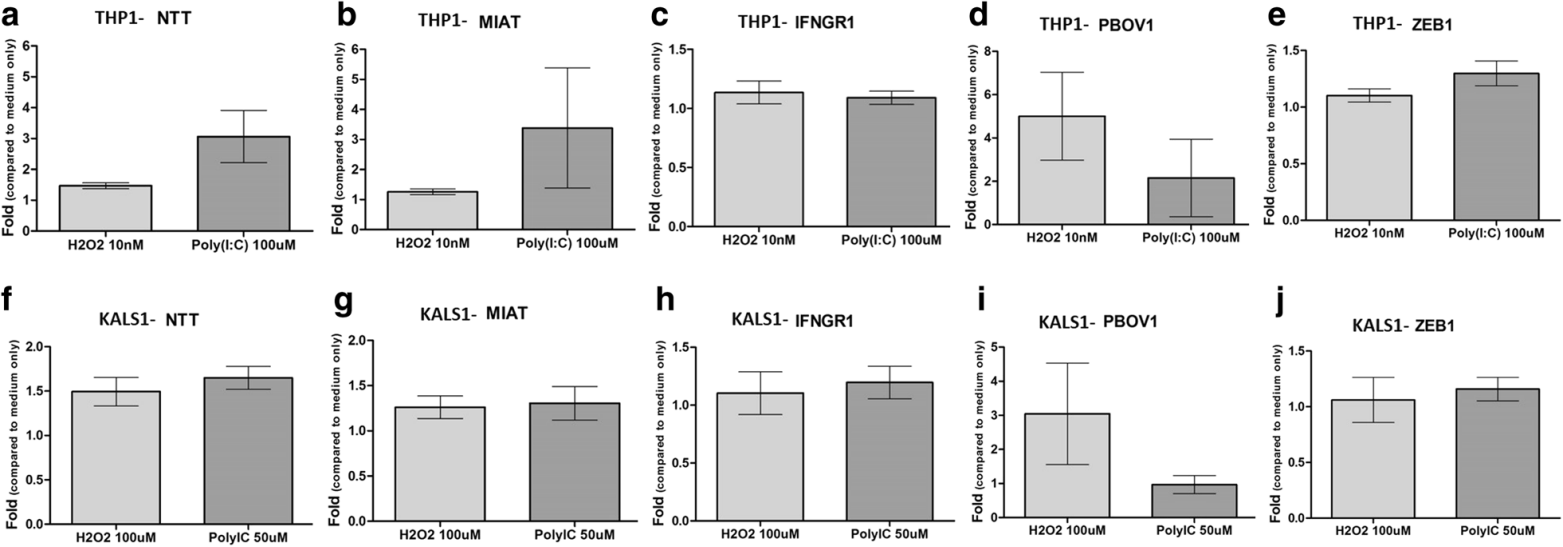
Upregulation of NTT and MIAT and potential downstream genes after peroxide or poly (I:C) stimulation in THP-1 and KALS1 cells compared to unstimulated cells. The expression levels of NTT (**a**) and MIAT (**b**) in THP-1 after 6 h stimulations. N = 6, bars represent mean ± SEM. The expression levels of *IFNGR1* (**c**) (N = 6), *PBOV1* (**d**) (N = 3) and *ZEB1* (**e**) (N = 6), bars represent mean ± SEM. The expression levels of NTT (**f**), MIAT (**g**), *IFNGR1* (**h**), *PBOV1* (**i**) and *ZEB1* (**j**) in KALS1 after 6 h stimulations. N = 3, bars represent mean ± SEM

Oxidative stress and recurrent herpes viral infection are known to contribute to ME/CFS pathology [28]. To investigate the potential effect of these stressors on the expression of NTT, MIAT and EMX2OS, we used human monocytic cell line THP-1 and glioma cell line KALS1 as models, and treated them with titrations of H_2_O_2_ (oxidative stress) or poly (I:C) (TLR3 agonist, mimicking herpes viral infection) for 6 h. While NTT and MIAT were upregulated after stimulations, EMX2OS could not be detected in both cell lines. In THP-1 cells, NTT expression level increased to a mean of 1.47- and 3.06-fold after stimulation with 10 nM H_2_O_2_ and 100 μM poly (I:C), respectively (Fig. 6a). Furthermore, MIAT level increased to a mean of 1.26- and 3.38-fold after THP-1 stimulation with 10 nM H_2_O_2_ and 100 μM poly (I:C), respectively (Fig. 6b). Higher expression levels of the potential NTT downstream gene *PBOV1* and the potential MIAT downstream gene *ZEB1* were also detected in THP-1 after stimulations with 10 nM H_2_O_2_ and 100 μM poly (I:C), respectively; however, the level of *IFNGR1* (another potential NTT downstream gene) did not show an obvious change after both stimulations (Fig. 6c–e). In KALS1 cells, NTT could be upregulated to a mean of 1.49-fold when H_2_O_2_ was titrated to 100 μM, and NTT level also increased to a mean of 1.65-fold after 50 μM poly (I:C) treatment (Fig. 6f). As for MIAT expression in KALS1, a 1.26-fold and a 1.31-fold increase were observed after 6 h 10 nM H_2_O_2_ and 100 μM poly (I:C) stimulations, respectively (Fig. 6g). The expression levels of potential NTT and MIAT downstream genes after H_2_O_2_ and poly (I:C) stimulations in KALS1 showed a similar pattern as those observed in THP-1 (Fig. 6h–j).

## Discussion

Emerging roles of very large (> 5 kb) lncRNAs in immune regulation and disease processes are being discovered [11]. Here we report the expression signature of ten lncRNAs NTT, NEAT1, MALAT1, TUG1, MIAT, His-1 RNA, GNAS1-AS, EMX2OS, CR933609 and AK124742 in PBMCs derived from ME/CFS patients. We have selected this screening panel according to their potential regulation of immune, stress, metabolic and neurologic responses, which have been hypothesized to be involved in ME/CFS pathogenesis. Nevertheless it is remarkable to find out on PCA plot that ME/CFS could be differentiated from healthy controls via this lncRNA profile. Among the ten lncRNAs, NTT, MIAT and EMX2OS expression levels explained the most variance between ME/CFS and controls. Further supporting their potential role in disease pathomechanism, higher NTT and EMX2OS levels were associated with more severe ME/CFS (Bell score < 30). Using expression of any two of these three lncRNAs (NTT, MIAT and EMX2OS) in discriminating ME/CFS from healthy had an AUC of 0.82 on ROC curve, suggesting a potential diagnostic value of these lncRNAs for ME/CFS.

Consistent with the hypothesis that disease pathology in ME/CFS could be driven by oxidative stress and viral infections, we found that NTT and MIAT levels in THP-1 and KALS1 cell lines could be increased after H_2_O_2_ or poly (I:C) stimulations, an expression pattern similar to those found in ME/CFS patients’ PBMCs. However, we do not know yet whether this lncRNA profile found in our study is specific for ME/CFS or can be found in other diseases involving immune dysregulation or oxidative stress, such as autoimmune diseases and cancer. It has been reported that MIAT levels could be upregulated in high glucose conditions and in lung cancer, and NTT expressions might be found in processes involving T cell activation [12, 17, 29]. Further comparing the PBMC expression signature of NTT, MIAT, and EMX2OS in ME/CFS with patients suffering from chronic fatigue due to autoimmune diseases or cancer is important to assess the lncRNA test specificity in diagnosing ME/CFS.

The mechanisms of NTT, MIAT and EMX2OS in ME/CFS pathogenesis require further investigations. In our study, we detected an association of higher level of *ZEB1*, a MIAT-regulated gene, with ME/CFS. Consistently, both THP-1 and KALS1 cell lines showed higher expression levels of MIAT and *ZEB1* after stimulation of poly (I:C), a synthetic analog of double strand RNA representing active viral infection, a potential trigger of ME/CFS. Zinc finger E-box-binding protein (ZEB) 1 has been reported to be a transcription factor recruiting repressor complex to suppress IL-2 activation in T cells [30]. Upregulation of *ZEB1* might be associated with response to chronic inflammation in ME/CFS. In non-small-cell lung cancer cell line, knockdown of MIAT resulted in decreased *ZEB1* expression, indicating cis-action of MIAT on regulating *ZEB1* [27]. Interestingly MIAT is involved in endothelial dysfunction as well, which is frequently observed in ME/CFS [17, 31]. For NTT, it has been proposed to exert its function on nearby genes due to its large size (17 kb) [12]. Several genes involving cell proliferation, apoptosis or inflammation, including *IFNGR1*, *PBOV1*, *TNFAIP3*, *HIVEP2*, *BCLAF1* and *MYB* are located close to the chromosome position of NTT [12]. We found no significant difference in *IFNGR1* and *PBOV1* expressions between ME/CFS and controls. However, a marked positive correlation between NTT and *IFNGR1* levels were observed in ME/CFS, not in controls. This observation suggests that the NTT/*IFNGR1* axis might play a subtle role in ME/CFS pathogenesis. Whether other downstream genes are affected by the upregulated NTT in ME/CFS and possibly play more important roles in disease pathobiology needs more experiments. Finally, according to lncrnadb, the lncRNA database, the expression level of EMX2OS under normal physiological condition is higher in brain, moderate in lymph nodes, and very low in leukocytes. Consistent with this, we could not detect EMX2OS in PBMCs of several individuals. However, most ME/CFS patients were found to have elevated EMX2OS in their PBMCs. The role of EMX2OS in PBMC is currently unclear, and the potential downstream gene *EMX2* usually expressed in CNS could not be detected in all study subjects [22]. Further EMX2OS overexpression experiment in THP-1 is underway in our lab to answer this question. Interestingly, E*MX2* upregulation was found in brain hypoxemia [32]. ME/CFS patients have broad decreases in cerebral blood flow, which may result in hypoxemia [33].

In addition to our study on using lncRNA signature as diagnostic marker for ME/CFS, profiles of blood mRNA expression and plasma metabolites have been suggested to show diagnostic values for ME/CFS [6, 7, 34]. As described by Kerr et al., there were 88 differentially expressed genes in ME/CFS required for diagnostic and prognostic grouping [34]. Furthermore, Naviaux et al. proposed an eight-metabolite set for diagnosing male ME/CFS, and a thirteen-metabolite set for diagnosing female ME/CFS [6]. Our results showed that a lncRNA expression panel composed of as few as three very large lncRNAs (NTT, MIAT, EMX2OS) may achieve a good diagnostic value and provide information about ME/CFS disease severity (NTT, MIAT).

## Conclusion

In conclusion, although the pathogenic mechanisms of very large lncRNAs in ME/CFS remains to be elucidated, we have first evidence that a lncRNA expression signature could be of diagnostic value in ME/CFS.
